# Photoluminescence enhancement by propagating surface plasmons confined in microresonators

**DOI:** 10.1038/s41598-026-53638-6

**Published:** 2026-06-17

**Authors:** Seina Miyamoto, Takeshi Komino

**Affiliations:** Graduate School of Science, University of Hyogo, Ako-gun, Hyogo, 678-1297 Japan

**Keywords:** Materials science, Optics and photonics, Physics

## Abstract

A number of studies of light-emitting devices have aimed to improve the luminescence efficiency, but the luminescence is affected by the presence of a metal layer. This is because electromagnetic waves propagating along the surface of a flat metal layer (or surface plasmon polaritons [SPPs]) generally deactivate excitons in emissive materials into the ground states. To overcome this problem, we propose a way of converting the deactivating character of SPPs into the opposite character to enhance the photoluminescence (PL). To achieve the PL enhancement, we localized SPPs to generate their standing waves in a whispering gallery mode microresonator. The standing waves were exploited to produce resonance between the oscillated electromagnetic fields of the SPPs’ standing waves and the transition dipoles of excitons so that the excitons can efficiently emit PL.

## Introduction

**Fig. 1:**
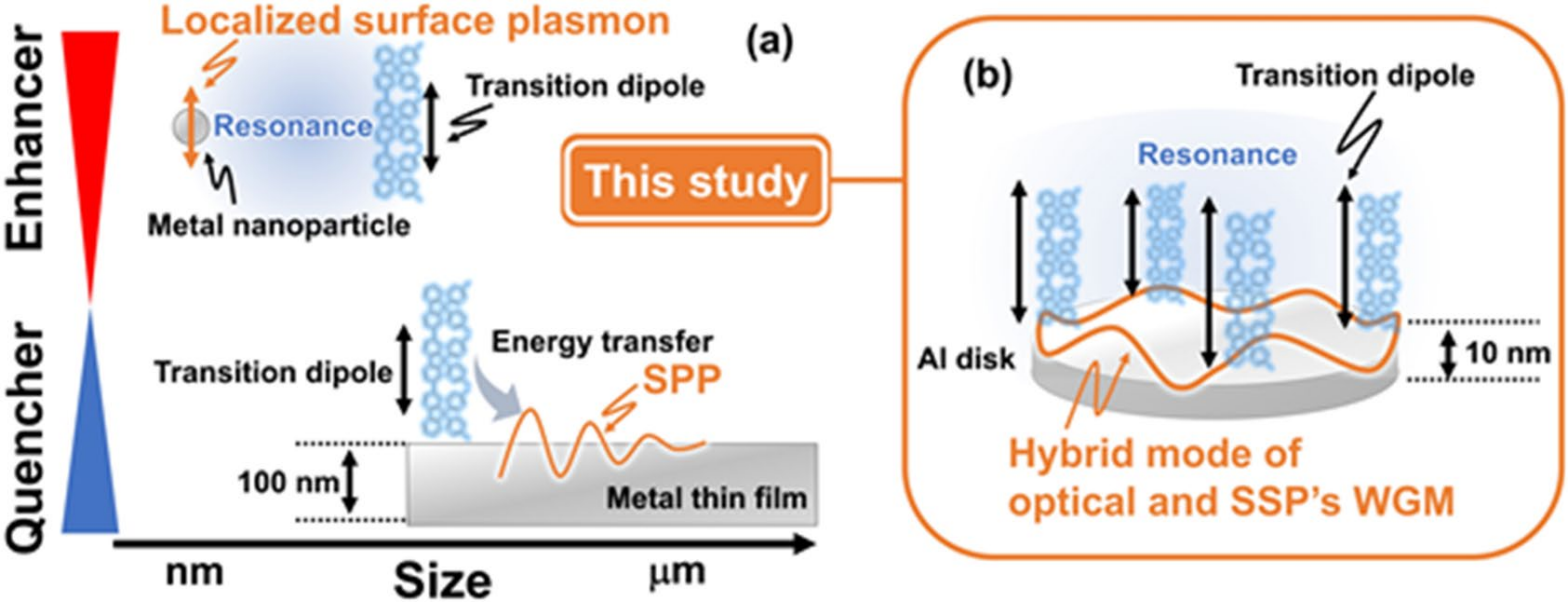
Effect of plasmons on photoluminescence (PL) enhancement/quenching. (**a**) Schematic of PL enhancement by localized surface plasmons and quenching by surface plasmon polaritons (SPPs). Generally, the magnitude of the quenching is larger for larger metal size on the micrometer scale. (**b**) The hybrid mode of the optical and plasmonic (SPP) whispering gallery modes (WGMs) produced in the microresonator can resonate with a transition dipole of the excitons near the metal surface to enhance the PL.

**Fig. 2:**
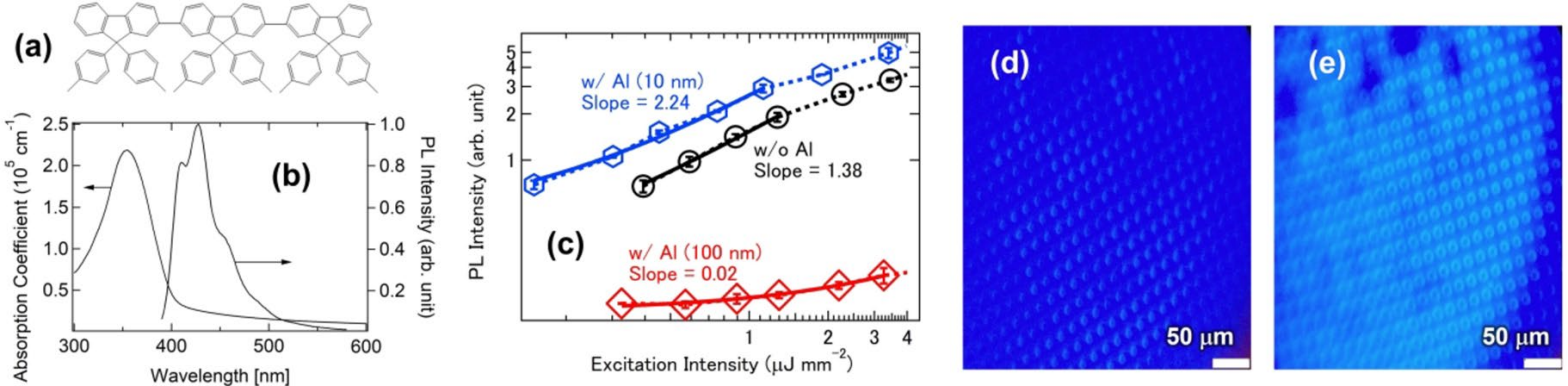
Molecular structure and PL characteristics of terfluorene. (**a**) Molecular structure of terfluorene. (**b**) Absorption and PL spectra of terfluorene thin films on Tempax glass substrates, where the terfluorene thicknesses were 50 nm (absorption) and 300 nm (PL). (**c**) PL intensity–excitation intensity characteristics of the resonators with 10- and 100-nm-thick Al layers and without an Al layer. The solid lines are linear fits. Influenced by optical setup, these slopes exhibit qualitative trends rather than absolute quantum efficiency (Table 1). PL emission images of the resonators with (**d**) 100- and (e) 10-nm-thick Al layers.

**Fig. 3:**
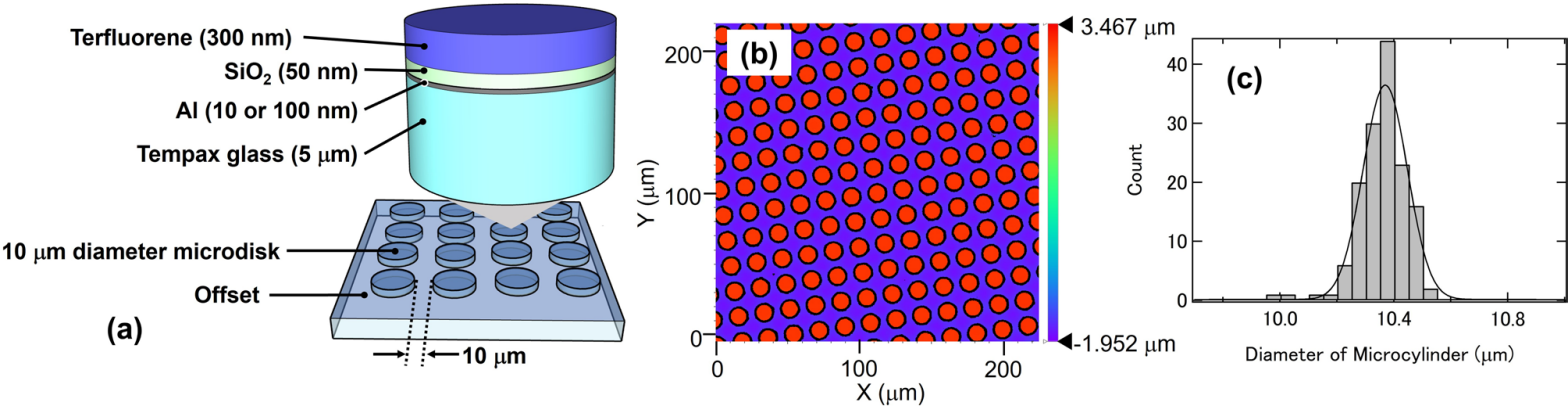
Characterization of the resonator structure. (**a**) Schematic of the resonator structure. The terfluorene layer is the part that enhances the PL, while the Al layer holds the SPPs. The WGM orbits at the edge of the resonator (see Fig. 2e). Offset indicates the base area without resonators. (**b**) Two-dimensional image and (**c**) histogram of the diameters of microcylinders obtained by analyzing the white light interferogram.

Over the past 60 years since the early studies of surface plasmons^1–3^, particularly since the discovery of light excitation of surface plasmons^4–6^, studies on light–matter interactions through surface plasmons have attracted much attention. While these studies of surface plasmons have provided a number of applications, such as sensors^7,8^ and enhancement of the photophysical properties (e.g., absorption^9^, fluorescence^10,11^, and Raman scattering^11–14^, surface plasmons are still sometimes out of control and occasionally have unintended effects on the material’s characteristics. Exciton quenching^15,16^ by surface plasmons is one example. The luminescence on metal surfaces has been studied since the 1960s.^17^ Specifically, when excitons are placed near metal surfaces, the excitonic energy is consumed to excite surface plasmon polaritons (SPPs)^18^ to deactivate the excitons into the ground states (Fig. 1a). The electrodes in a number of optoelectronic thin film devices are made of metals, and therefore this type of quenching often occurs. In organic light-emitting diodes, the quenching by SPPs accounts for several tens of percent of the total power dissipation of the generated excitonic energies^19^. Therefore, an ideal strategy to improve the luminescence efficiency is to somehow exploit SPPs for luminescence enhancement. Okamoto et al.^20^ succeeded in extracting the energy of SPPs, which is coupled to excitons confined in quantum wells, to air as a radiation mode to enhance the PL. In the structure, the non-flat metal/semiconductor interface plays a crucial role in the light outcoupling of the SPPs. This is because it is difficult to couple the SPPs out of the structure when the interface is completely flat.^21^(Even when SPPs can couple to a radiation mode as surface plasmon-coupled emission^22,23^, the PL intensity generally does not dramatically change.) Even with a flat interface, however, PL enhancement is expected by another mechanism, e.g., electromagnetic field enhancement around excitons by SPPs. Here, it should be noted that the PL enhancement we mean is not light amplification^24^ but enhancement of the spontaneous emission. Such PL enhancement requires standing waves of SPPs. In other words, we need to localize SPPs into a metallic structure. Furthermore, if the structure can be fabricated from a macrostructure rather than from a conventional nanostructure, it will be of significance for application to metal electrode layers for the enhancement of the luminescence in optoelectronic devices. We recently succeeded in localizing SPPs in a 20-µm-diameter circular microresonator^25^. Using the whispering gallery mode (WGM)^26–28^ of a SPP in very thin metal films^29,30^, which was generated in a 10-nm-thick Al thin film in our case, the PL enhancement of 2,7-bis[9,9-di(4-methylphenyl)-fluoren-2-yl]-9,9-di(4-methylphenyl)fluorene (terfluorene, Fig. 2a) balanced the quenching effect. Hence, PL enhancement is currently very close to overcoming quenching, although there is still room for improvement of the resonator structure. Accordingly, in this study, we spatially separated a part of the enhanced PL from a part of the holding SPPs in a WGM resonator to greatly suppress the effect of quenching on the net PL enhancement. The resonator structure is shown in Fig. 3a. As a result, we discovered that the PL enhancement overcomes quenching to improve the PL quantum yield (PLQY) even by a flat and large (10-µm-diameter) Al film as shown in Fig. 1.

**Table 1:** Photoluminescence quantum yield (PLQY) of each structure.

Al thickness (nm) SiO_2_ thickness (nm) Al thickness (nm)	PLQY
Layer (s) on flat substrates	
–	0.65 ± 0.02 ^*a*^
10	0.35 ± 0.01 ^*b*^
100	0.36 ± 0.01 ^*b*^
With 10 μm diameter WGM resonators	
–	0.69 ± 0.05 ^*c, d*^
10	0.85 ± 0.01 ^*b, d*^
100	0.15 ± 0.02 ^*b, d*^

^*a*^ The sample without SiO_2_. ^*b*^ The SiO_2_ thickness was 50 nm. ^*c*^ The SiO_2_ thickness was 60–150 nm. ^*d*^ The influence of the offset area on the directly measured PLQY was corrected.

## Results

### Improvement of PLQY

The absorption and PL spectra of the terfluorene thin film fabricated on a planar glass substrate (terfluorene/glass) are shown in Fig. 2b. Strong π–π* absorption appeared around 350–370 nm. We used 350 nm for the light excitation of the terfluorene layers in the PL characterizations. The PL band appeared at 400–600 nm with peaks and shoulders at 410, 427, 452, and 490 nm. The spectral shape was independent of the layer stacking structure and the presence/absence of the resonator structure.

The PLQY of each structure is given in Table 1. The PLQY of the simple planar terfluorene/glass structure was 0.65 ± 0.02, which is consistent with the literature^31^. We hereafter consider this value to be the control (i.e., the ordinary PLQY of terfluorene thin films). Using the WGM resonator containing a 10-nm-thick Al layer, the PLQY was enhanced to 0.85 ± 0.01. This value was calculated by $\:{PLQY}_{disk}={\left(\frac{{S}_{disk}}{{S}_{disk}+{S}_{offset}}\right)}^{-1}\left({PLQY}_{total}-{PLQY}_{offset}\frac{{S}_{offset}}{{S}_{disk}+{S}_{offset}}\right)$, where $\:S$ is a surface area. The subscripts total, disk, and offset denote the whole area of the sample, the inner area of the disk, and the area without resonators (we denote this area the offset area, Fig. 3a), respectively. The above equation corrects for the influence of the offset area on the directly measured PLQY to calculate $\:{PLQY}_{disk}$. Namely, the above PLQY of 0.85 is $\:{PLQY}_{disk}$. $\:{PLQY}_{total}$ is the PLQY obtained directly in the measurement, and $\:{PLQY}_{offset}$ was independently measured to be 0.35 ± 0.01 using the no-resonator structure of glass/Al (10 nm)/SiO_2_ (50 nm)/terfluorene (300 nm). The difference between $\:{PLQY}_{disk}$ (0.85, with resonator) and $\:{PLQY}_{offset}$ (0.35, without resonator) indicates the importance of the presence of the resonator on the PL enhancement. We ascribe the PL enhancement to the electromagnetic resonance of the terfluorene excitons with the hybrid mode^32,33^ of the plasmonic^34^ and optical WGMs (Fig. 1b).

To verify the effect of the hybrid mode on the electromagnetic resonance, we assessed the slopes of the PL intensity–excitation intensity curves (Fig. 2c). The slopes include the factors of the optical geometry in the measurement setup and the photophysical properties (e.g., concentration quenching). Therefore, they cannot be directly used for evaluating the PLQYs. However, the slopes are roughly proportional to the PLQYs. The slope in the resonator with 10-nm-thick Al was 2.24, while that without Al was 1.38, meaning that the PL was enhanced by the presence of the hybrid mode.

### Origin of the PL enhancement

**Fig. 4:**
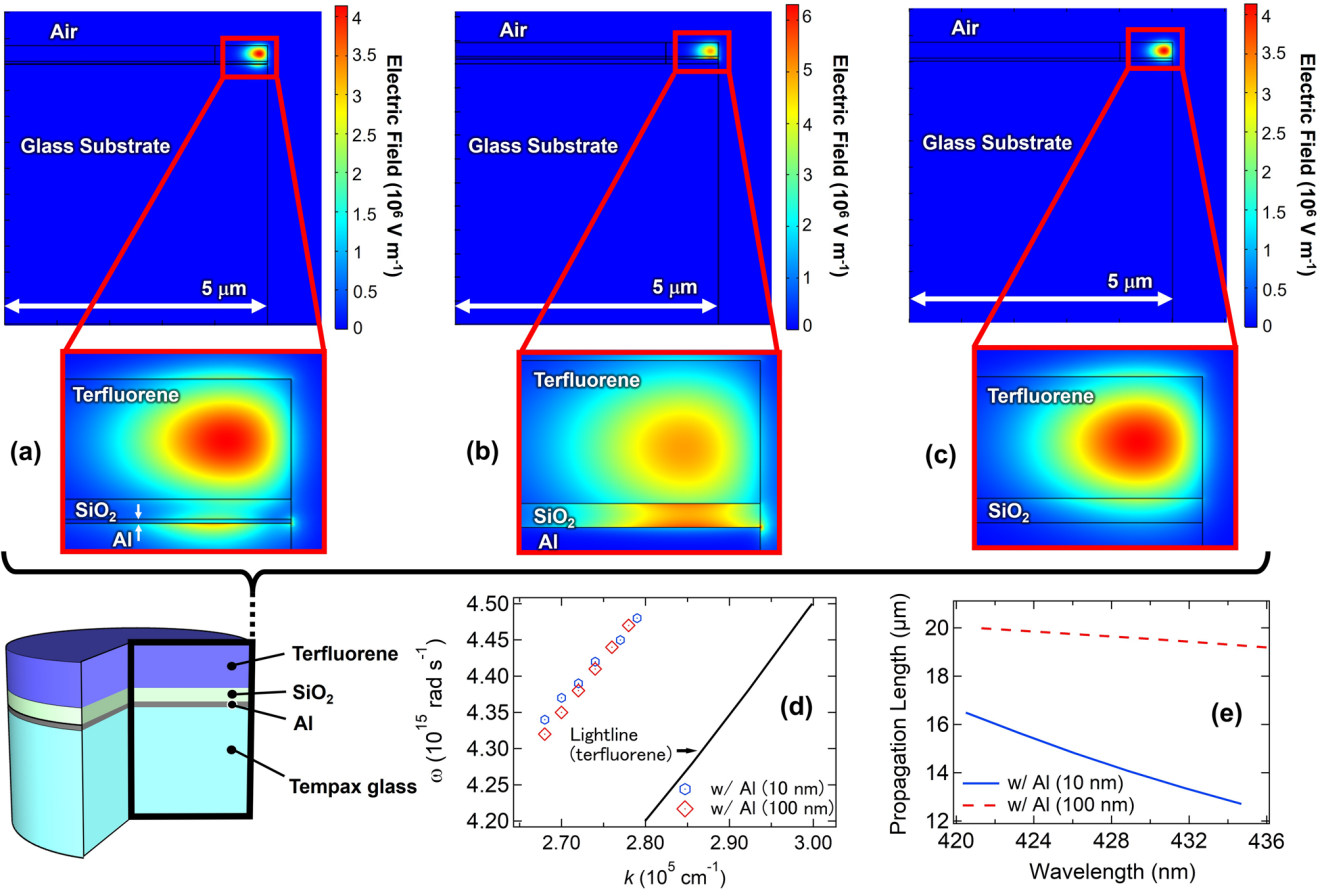
Wave optics simulation results. Spatial distributions of the electric fields in the resonators (**a**) with a 10-nm-thick Al layer, (**b**) with a 100-nm-thick Al layer, and (**c**) without an Al layer. These images show the two-dimensional planes of the cross sections of the resonators from the centers to the side edges. (**d**) Dispersion relations for the resonators with 10- and 100-nm-thick Al layers. $\:k$ denotes the effective refractive wavevector. The lightline for terfluorene is also plotted as the standard to compare the dispersion relations of the resonators. (**e**) Propagation lengths for the resonators with 10- and 100-nm-thick Al layers.

A wave optics simulation showed that the evanescent electric fields of the plasmonic WGM appeared at the upper and lower sides of the 10-nm-thick Al layer (Fig. 4a), and that the upper electric field combined with the optical WGM, which is confined in the vicinity of outer edge of the terfluorene thin film microdisk. Considering that these two modes simultaneously appear in the same eigenmode, the eigenmode can be considered to be the hybrid mode of the plasmonic and optical WGMs. The electric field of the hybrid mode can resonate with the transition dipoles of the terfluorene excitons, giving rise to the PL enhancement.

The hybrid mode appeared even in another resonator containing a 100-nm-thick Al layer (Fig. 4b). This was the same as the above case for 10-nm-thick Al. The dispersion relations for the resonators containing 10- and 100-nm-thick Al layers were almost the same, as shown in Fig. 4d. This means that the orbiting modes in both resonators are present in almost the same media (mainly terfluorene and SiO_2_). However, the mode in 100-nm-thick Al does not necessarily produce PL enhancement. Cautiously comparing both simulated electric field distributions (Fig. 4a and b), the SPPs in 100-nm-thick Al selectively accumulate the electric field in the vicinity of the Al surface. This differs from the case of 10-nm-thick Al, where the electric field is accumulated in the terfluorene layer. Thus, compared with that in the 10-nm-thick Al layer, the electric field of the SPPs in the 100-nm-thick Al layer is relatively large so that the SPPs require large excitation power, which stems from the energy transfer from the terfluorene excitons (Fig. 1a). Therefore, it is not surprising that the terfluorene excitons are rapidly quenched. This prediction is in good agreement with the result of the PLQY measurements. The PLQY of the WGM resonator containing the 100-nm-thick Al layer was 0.15 ± 0.02, which was significantly smaller than the control (0.65). This is consistent with the PL intensity–excitation intensity characteristics (Fig. 2c). The slope of the PL intensity–excitation intensity curve for the resonator with 100-nm-thick Al (0.02) was smaller than that for the resonator with 10-nm-thick Al (2.24). PL microscope images supported the interpretation. In the case of 100-nm-thick Al (Fig. 2d), the bright emission rapidly decayed and did not orbit the whole of the outer edge of the resonators, suggesting that the terfluorene excitons were rapidly quenched by the SPP’s excitation before formation of a hybrid WGM and subsequent PL enhancement. This was different from the case of 10-nm-thick Al (Fig. 2e), where ring-shaped PL emission near the outer edge of each resonator was observed, demonstrating the PL enhancement by the hybrid WGM. It should be noted that it is difficult for the simulation to predict whether or not PL enhancement occurs because the simulation adopts wave optics rather than quantum optics, which rules out the interplay between the hybrid WGM and excitons. In other words, while the distinction of PL enhancement or quenching is governed by the power balance between the excitons and hybrid mode, the simulation does not estimate this balance. This is consistent with the analysis of the propagation length of the SPPs (Fig. 4e). The propagation length for the resonator with 100-nm-thick Al was longer than that for the resonator with 10-nm-thick Al, indicating that the SPP in the resonator with 100-nm-thick Al more easily survives than that in the resonator with 10-nm-thick Al. However, the survived mode in the experiment with 100-nm-thick Al easily decays because the excitons are readily quenched by consuming much of the power to oscillate a larger number of free electrons (or SPPs) than in the resonator with 10-nm-thick Al.

In the PL enhancement, the contribution of the plasmonic WGM is particularly important because this contribution is unpredictable from the conventional SPPs’ behavior as follows. The plasmonic WGM travels in the micrometer-scale Al disk, and thus the plasmonic WGM is categorized into SPPs. Generally, SPPs quench excitons when the excitons are placed near the metal surface because SPPs absorb the excitonic energy and then escape from the exciton site (Fig. 1a). As a result of the quenching by the SPPs, $\:{PLQY}_{offset}$ (0.35) was much smaller than the control (0.65). However, the SPPs in the resonator with 10-nm-thick Al form standing waves, or the plasmonic WGM, to be localized in the vicinity of the terfluorene excitons, resulting in a contribution to the PL enhancement. The mechanism behind the PL enhancement is similar to a well-known metal-enhanced fluorescence^35^ including localized surface plasmon resonance (Fig. 1a), but the material size is different. In contrast to a localized surface plasmon confined in a metal nanostructure, the plasmonic WGM realizes PL enhancement in the metal macrostructure (Fig. 1a and b).

We emphasize that the contribution of the plasmonic whispering gallery mode (WGM) rather than that of the optical WGM is important for PL enhancement. The optical WGM can also enhance the PL. In fact, $\:{PLQY}_{disk}$ of the glass microcylinder (5 μm)/SiO_2_ (60–150 nm)/terfluorene (300 nm) structure (i.e., without SPPs) was 0.69 ± 0.05 independent of the SiO_2_ thickness, which was larger than the control (0.65). This result was supported by a wave optics simulation, as shown in Fig. 4c. The electric field of the optical WGM is broadly distributed in the terfluorene thin film, which can resonate with the terfluorene excitons. However, the PLQY of 0.69 is insufficient to account for the whole of the PL enhancement in the resonator including the 10-nm-thick Al layer ($\:{PLQY}_{disk}$ = 0.85). This suggests that the PL enhancement in the resonator including the Al layer does include the contribution of the plasmonic WGM to the resonance between the hybrid mode and excitons. To reveal the detail of the mechanism behind the PL enhancement (the Purcell factor, the dipole radiation from SPPs, and other possible mechanisms), transient PL measurements will be conducted in the future.

**Fig. 5:**
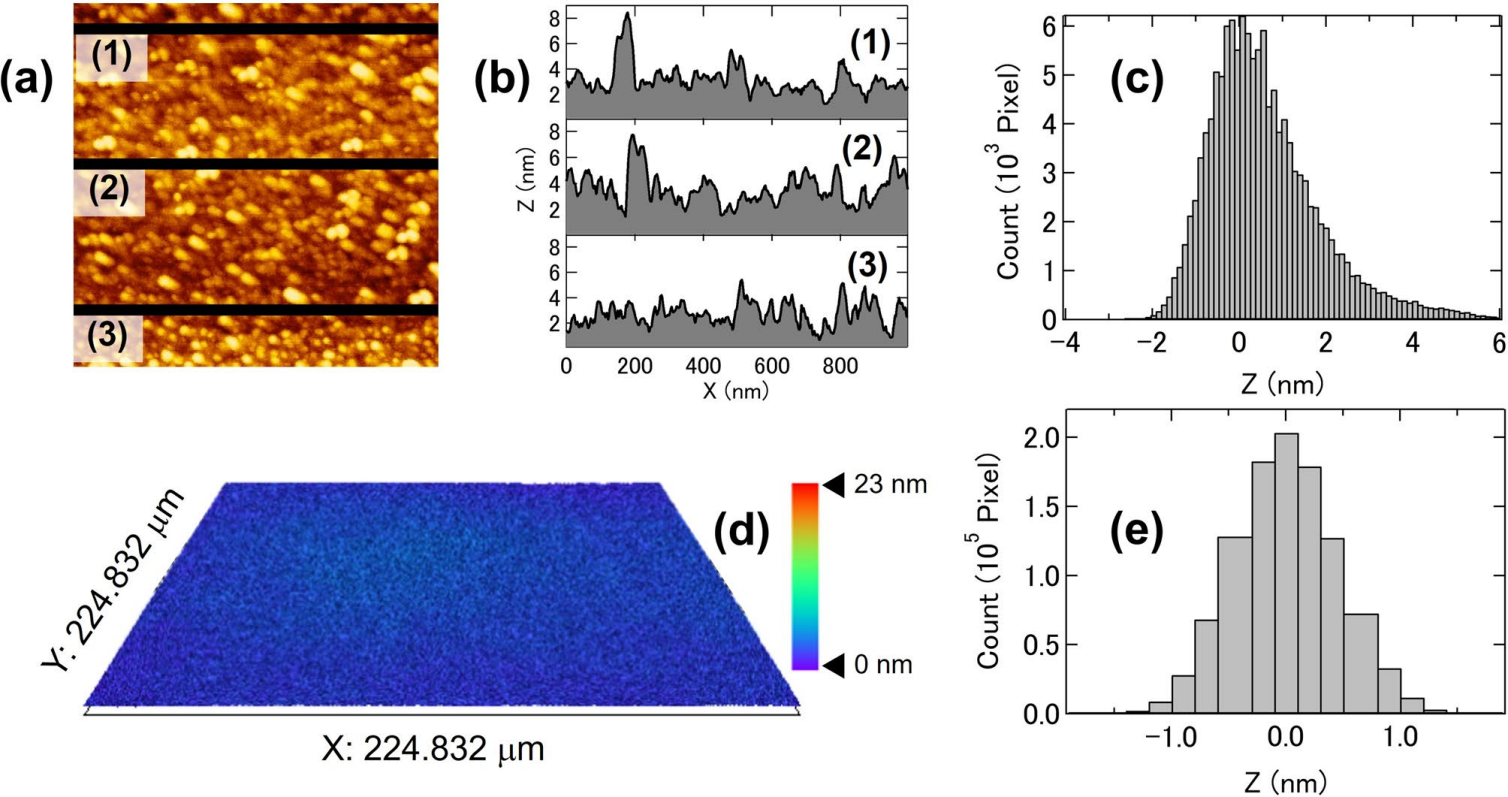
Surface morphology of a 10-nm-thick Al thin film. (**a**) Atomic force microscopy (AFM) image of a 10-nm-thick Al thin film deposited on a planar Tempax substrate and (**b**) the cross-sectional profiles of the AFM image. The numbers 1–3 denote the positions in the AFM image (a). (**c**) Histogram of the *Z* values of the AFM image (a). (**d**) Three-dimensional image of the identical 10-nm-thick Al thin film obtained by white light interferometry. (**e**) Histogram of the *Z* values in the interferometry image (d).

We briefly discuss another possibility that the distinction between the PL enhancement and quenching arose not from the presence of the hybrid mode, as described above, but from the difference in the chemical compositions. To investigate whether the Al layers were composed of metallic Al or of the oxide, we measured the sheet resistances. We found that the sheet resistances of 10- and 100-nm-thick Al were 71 and 6.0 Ω/sq, respectively. Given that the thickness of the natural oxide (Al_2_O_3_) layer is independent of the Al thickness, the resulting sheet resistances lead to an Al_2_O_3_ thickness of 1.6 nm, which is in good agreement with reported thicknesses [1–3.5 nm^36,37^]. This result means that 84% of the 10-nm-thick Al layer was indeed composed of metallic Al. Therefore, the hybrid mode can be generated even in 10-nm-thick Al, and it can contribute to the PL enhancement. In addition, the Al film surfaces were smooth so that the roughness could not create a scattering center. The surface roughness was assessed by atomic force microscopy (AFM) and white light interferometry for a small area (1 μm × 1 μm) and a large area (larger than 200 μm × 200 μm), which was larger than the size of the microresonators. An AFM image of the 10-nm-thick thin film and cross-sectional profiles of three positions in the AFM image are shown in Fig. 5a and b, respectively. Although grains were observed, the Al thin film surface was smooth. The histogram of the *Z* values in the AFM image is shown in Fig. 5c. The estimated root-mean-square roughness was 1.3 nm. The smoothness was guaranteed even in the large area, as shown in the white light interferometry image (Fig. 5d) and the histogram of the *Z* values (Fig. 5e). The estimated root-mean-square roughness was 0.7 nm, which is consistent with that in the AFM analysis. Therefore, we consider that the surface roughness over the Al layer surface in the resonators is included in the range from 0.7 to 1.3 nm. This roughness is two orders of magnitude smaller than the wavelength of the SPPs.

## Discussion

The PLQY of terfluorene was enhanced from 0.65 to 0.85 by the resonances among the plasmonic WGM, optical WGM, and terfluorene excitons. The plasmonic WGM was generated in the micrometer-scale resonator, so the generated plasmon is categorized into a SPP. Such a SPP generally quenches excitons, which are placed near metal surfaces, by absorbing the excitonic energy and escaping from the site. However, the SPP causes the WGM to be localized in the vicinity of excitons to realize PL enhancement through the resonance between the excitons and the hybrid mode of the plasmonic and optical WGMs. This reveals that PL enhancement, like localized surface plasmon resonance, is feasible even in the micrometer-scale macrostructure. We expect that even the metallic electrodes used in organic optoelectronic devices (e.g., organic light-emitting diodes), which generally cause quenching, can enhance the quantum efficiency of luminescence by such a plasmonic WGM.

## Methods

### Fabrication of resonators

Terfluorene, Al wire (99.99%) for the vacuum deposition source, and the SiO_2_ sputter target (99.99%) were purchased from Lumtec, Nilaco, and K’s Tech, respectively, and they were used without further purification. The plasmonic WGM microdisk, as shown in Fig. 1b, was fabricated on 10-µm-diameter and 5- µm-high glass microcylinders, which were two-dimensionally aligned on a surface of a Tempax glass substrate (63.5 mm × 63.5 mm × 1.1 mm, Matsunami Glass) to form a square grid pattern with a distance of 10 μm between adjacent microcylinders. The white light interferometry image of the fabricated Tempax microcylinders is shown in Fig. 3b. The image was acquired with a white light interferometer (VS-1000, Hitachi) with an objective lens magnification of 50×. To fabricate the microcylinders, the Tempax glass substrate surface was first cleaned by sonication using a diluted detergent (Cica Clean LX-II, Kanto Chemicals) in water, followed by rinsing with water, acetone, and 2-propanol, ultraviolet–ozone treatment, and coating with a 200-nm-thick Cr layer by vacuum deposition. The Cr layer supported a photoresist layer (AZP4620), which was spin coated at 500 rpm for 5 s and 2000 rpm for 20 s. After prebaking at 110 °C for 100 s, the photoresist was exposed to ultraviolet light (27.5 mW cm^− 2^ for 17 s) through a custom-made photomask (Equa) to shade a 10-µm-diameter spot array on the resist layer using a mask aligner (PEM-800, Union Optical). The processed substrate was then immersed in a developer (NMD-3, Tokyo Ohka Kogyo) for approximately 5 min to expose the Cr layer by dissolving the light-exposed resist. After rinsing in water for 10 min and baking at 130 °C for 3 min, the substrate was immersed in a Cr etchant (SEK-1, Konan Muki) for 3 min to remove the exposed Cr layer. The substrate was then rinsed in water for 10 min. The exposed glass surfaces were cleaned by an oxygen plasma ashing reaction using a dry etching machine (FA-1, Samco) with a radio frequency power of 20 W for 20 s and etched by CF_4_ plasma using an inductively coupled plasma-reactive ion etching machine (RIE-101iPH, Samco) with a bias power of 100 W, radio-frequency power of 300 W, CF_4_ pressure of 3 Pa, and CF_4_ flow of 20 sccm. The residual photoresist and Cr were removed by immersion in a piranha solution at 80 °C for approximately 30 min and a Cr etchant for approximately 3 min, respectively. We confirmed that each microcylinder was successfully fabricated with the desired diameter and height by white light interferometry. The two-dimensional images and the histogram of the diameters are shown in Fig. 3b and c, respectively. The histogram was well fitted by a Gaussian function, and the dispersion of the fit was 0.11 μm. The 10- or 100-nm-thick Al layer was thermally deposited on the top surface of the glass microcylinder, followed by deposition of a 50-nm-thick SiO_2_ layer, which was fabricated by radio-frequency magnetron sputtering with an Ar flow of 1.0 sccm under pressure of 0.3 Pa. The 300-nm-thick terfluorene layer was thermally deposited on the Al/SiO_2_ stacked layers under a vacuum of < 1 × 10^− 3^ Pa to form a 10-µm-diameter WGM resonator with the structure of glass microcylinder (5 μm)/Al (10–100 nm)/SiO_2_ (50 nm)/terfluorene (300 nm). Reference structures were also fabricated by changing Al to SiO_2_ to form a glass microcylinder (5 μm)/SiO_2_ (60–150 nm)/terfluorene (300 nm) structure, by replacing the microcylinders with planer glass substrates to form a planer glass/Al (10–100 nm)/SiO_2_ (50 nm)/terfluorene (300 nm) structure, and by removing the microcylinders and Al/SiO_2_ layers to form a simple planer glass/terfluorene (300 nm) structure.

### PLQY measurements

The PLQY of each structure was measured by a fluorescence spectrometer (F-7000, Hitachi) with an excitation wavelength of 350 nm and entrance and exit slit widths of 5 nm. The samples were placed at the sample space so that the thin film side faced the center of an integrating sphere, and thus the excitation light was incident from the terfluorene thin film side.

### Absorption and PL spectra of terfluorene

The absorption spectrum of terfluorene was acquired with the simple planer glass/terfluorene structure using a spectrometer (UV-2400 PC, Shimadzu) with a slit bandwidth of 1.0 nm. The photoluminescence (PL) spectrum was acquired by a fluorescence spectrometer (F-7000, Hitachi) with an excitation wavelength of 350 nm and entrance and exit slit widths of 5 nm.

### PL imaging and PL intensity–excitation intensity characteristics of the resonators

The PL microscope images were acquired with an image sensor (DP74, Olympus) through an objective lens with a magnification of 40×. The WGM resonators were mounted on the sample stage of the microscope (MMSP, Olympus) so that the resonators faced the objective lens and excited at 365 nm from the oblique direction (the objective lens side) using an ultraviolet light lamp (SLUV-6, As One).

Using the same detector system, the PL intensity–excitation intensity characteristics were acquired, replacing the excitation light source from the ultraviolet lamp with a 5-ns pulsed Nd-YAG laser with an excitation wavelength of 355 nm. The excitation light was incident from the terfluorene thin film side. The excitation intensity was controlled by a rotation-type neutral density filter. The excitation intensity was lowered so that light amplification (or lasing) could not occur. The shapes of the PL spectra were independent of the excitation intensities. The PL intensities at the maximum PL peak were plotted to obtain the PL intensity–excitation intensity characteristics (Fig. 2c).

### Wave optics simulations

To compare the electric field distributions among the glass microcylinder (5 μm)/Al (10–100 nm)/SiO_2_ (50 nm)/terfluorene (300 nm) structures, wave optics simulations were performed with a two-dimensional axisymmetric model^38^. The simulations were performed by the finite-element method with a maximum element size of 0.01 μm for the near edges of the resonators and 10 μm for the other areas using a combination of COMSOL Multiphysics (version 6.2) and the Wave Optics Module (version 6.2). The refractive indices of air, Tempax (the substrate and microcylinder), terfluorene, and SiO_2_ were set to 1.0, 1.5 (the data from the manufacturer), 2.0^39^, and 1.5^40^, while their extinction coefficients were set to 0. The optical constants, including the wavelength dispersions of Al, were set considering the literature^41^. The details of the simulations have been described elsewhere^25^.

To obtain the dispersion relations and propagation lengths, we extracted the real and imaginary parts of the effective refractive indices ($\:{N}_{eff}$) from the eigen frequency ($\:f$). The real part is given by $\:mc/\left(2\pi\:R\mathrm{R}\mathrm{e}\left(f\right)\right)$, where $\:m$ is the mode index, $\:c$ is the light velocity in vacuum, and $\:R$ is the WGM radius. The effective wavevector ($\:k$) in the dispersion relation was calculated by $\:k=\mathrm{R}\mathrm{e}\left({N}_{eff}\right)2\pi\:/\lambda\:,$ where $\:\lambda\:$ is the eigen wavelength in vacuum. The imaginary part is $\:\mathrm{I}\mathrm{m}\left(f\right)\lambda\:/c$. The propagation length was calculated by $\:{\left\{2\left(\mathrm{I}\mathrm{m}\left({N}_{eff}\right)2\pi\:/\lambda\:\right)\right\}}^{-1}$.

### Sheet resistance and surface roughness of the Al thin films

Sheet resistance measurements of the Al thin films vacuum deposited on Tempax glass substrates were performed by a four-probe method using a four-wire cable (SR4-S, Astellatech) and a direct current voltage current source/monitor (6240B/ADCMT).

The surface roughness was evaluated by white light interferometry and AFM using a white light interferometer (VS-1000, Hitachi) with an objective lens magnification of 50× and an atomic force microscope (AFM 5500 M II).

## Data Availability

The data that support the findings of this study are available from the corresponding author upon reasonable request.
